# Investigating host plant association, calling activity, and sexual dimorphism in Indian *Gryllacropsis* sp. (Orthoptera: Anostostomatidae)

**DOI:** 10.1002/ece3.6819

**Published:** 2020-10-15

**Authors:** Manisha Tomar, Swati Diwakar

**Affiliations:** ^1^ Department of Environmental Studies University of Delhi Delhi India

**Keywords:** bioacoustics, invertebrates, resource selection function, seasonal calling, wetas

## Abstract

Both sexes of Indian weta *Gryllacropsis* sp. communicate acoustically. Females lack an external ovipositor making it difficult to differentiate between the sexes in the field. There is limited ecological information on the species as it is found high up on the trunks of evergreen trees, well camouflaged, and active only at night. The present study was conducted to gain ecological information on this little known yet intriguing species. We tested the hypotheses that (a) calling activity of Indian weta is uniformly distributed throughout the year and (b) there is no difference in morphometric measurements between the sexes. The study was conducted in Bhagwan Mahavir Wildlife Sanctuary and Mollem National Park, Goa, India. Visual scanning of tree trunks followed by vegetation sampling, psychoacoustic sampling, and morphometric analyses was carried out. Resource selection function values, obtained for a total of 52 tree species from 1984 individuals, were less than 0.1 for all plant species indicating no preference by the wetas. Peak calling activity was observed in the month of November (Rayleigh's test, *Z* = 7.90, *p* < .01). Discriminant function analysis on morphometric characters of males and females (Wilk's *λ* = 0.32 approx. *F* (4, 21) = 11.24 *p* < .0001, classification accuracy = 96.15%) provided clear distinction between males and females. Contribution of body weight was significant (standardized canonical discriminant function coefficients = +1) and could be used for identification of sexes in the field. These polyphagous insects provide insights on understanding ecological specialization due to host plant association, signal evolution, and mating behavior.

## Graphical abstract

The study investigated host plant association and calling activity of an Indian weta (*Gryllacropsis* sp.). The study also investigated sexual dimorphism in morphometric characters which could be used for identification of sexes in the field in the absence of external ovipositor in female wetas.

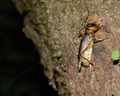

## 1 INTRODUCTION

**FIGURE 1:**
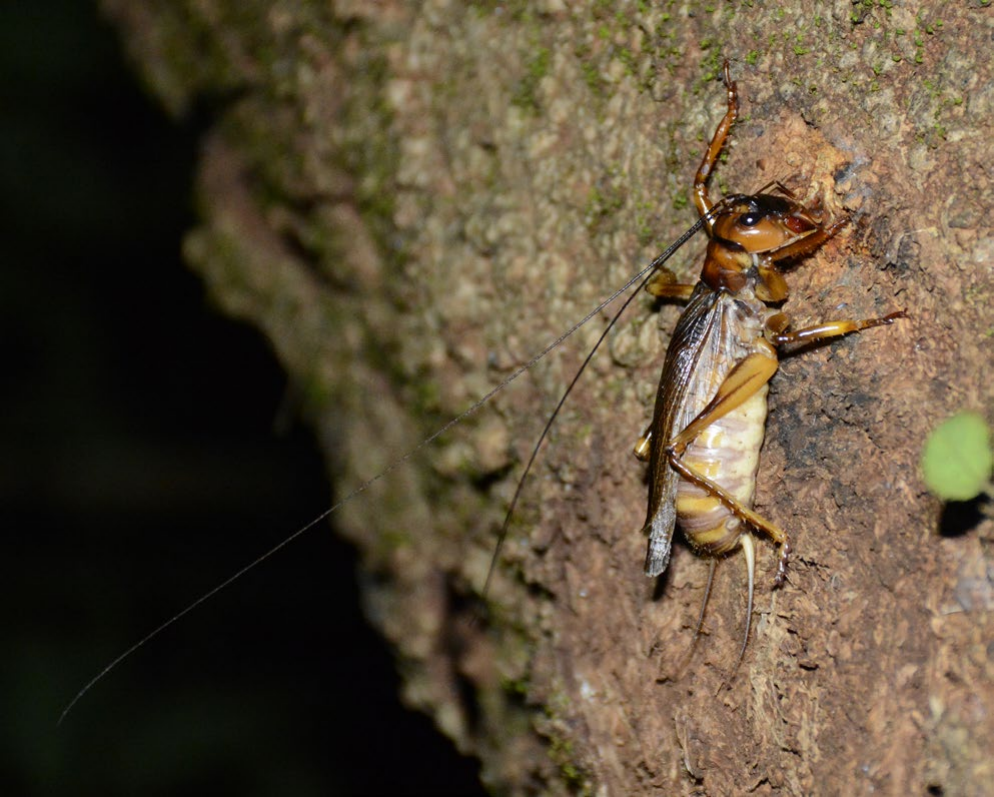
*Gryllacropsis* sp. in its natural habitat on the trunk of an evergreen forest tree

*Gryllacropsis* sp. belongs to the family Anostostomatidae (Superfamily: Stenopelmatoidea) in the suborder Ensifera (Order: Orthoptera) (Johns, 1997). Wetas are found predominantly in New Zealand but are distributed across Africa, Australia, South and Central America, and Asia (Gorochov, 2001). Wetas are among one of the ancient (Carboniferous) insects and are also considered as the largest among the insects (McIntyre, 2001; Wey & Kelly, 2019). Wetas are unique due to their morphology characterized by large body, thread‐like antennae that can extend up to two or three times of their body size, and menacing jaws that add to their fierce looks (Gibbs, 1998; Green & Ramsay, 2003) (Figure 1).

**FIGURE 2:**
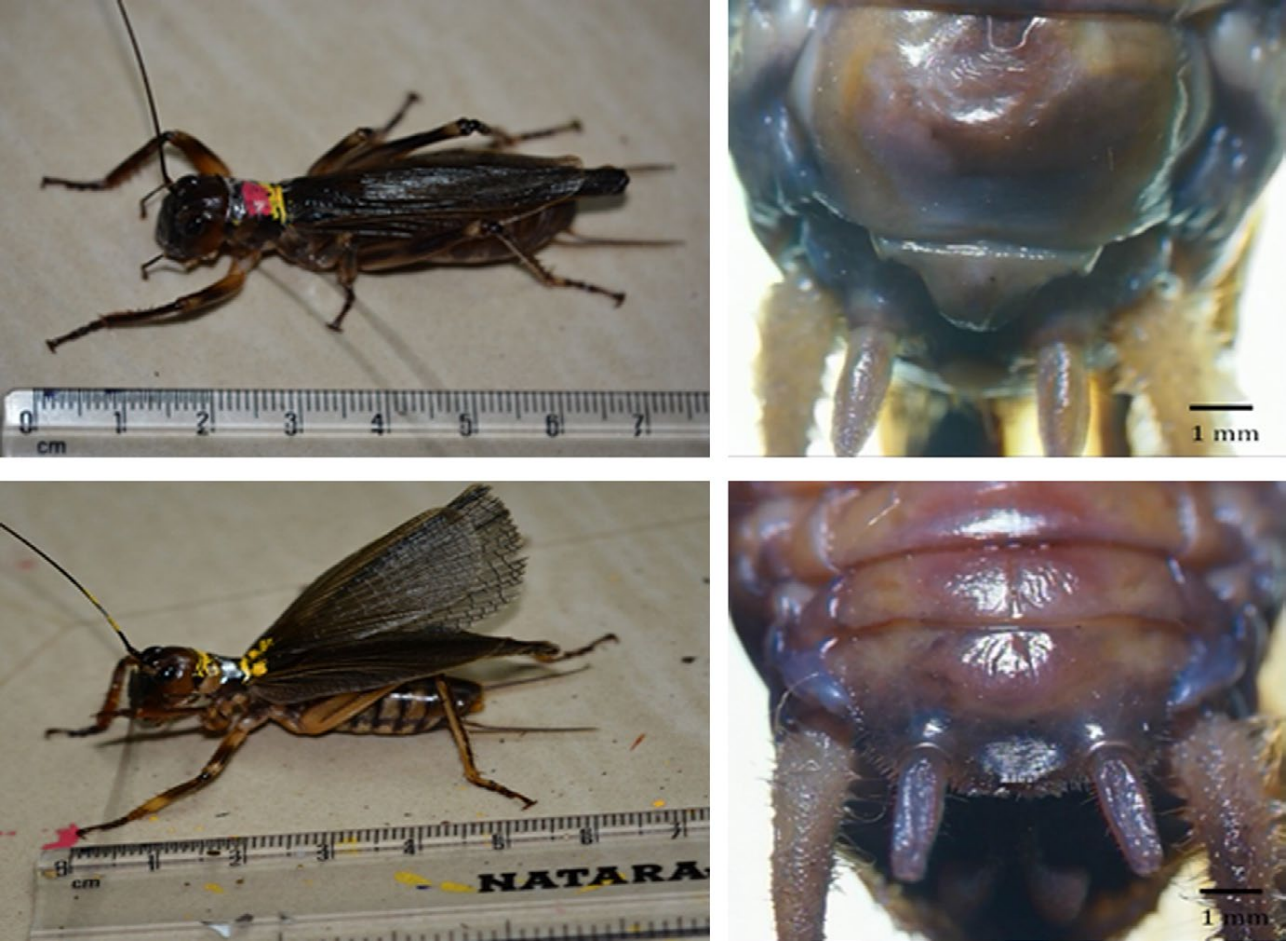
External morphology of *Gryllacropsis* sp. Upper trace showing adult female and external genitalia of female; lower trace showing adult male and external genitalia of male. The pink and yellow colors on the pronotum of wetas were due to paint markers used and are not the natural coloration of the wetas

*Gryllacropsis* sp. endemic to biodiversity hotspot of Western Ghats (Johns, 1997) is nocturnal and arboreal, and there is limited ecological information available on these organisms. Studies on the Indian weta have reported that *Gryllacropsis* sp. called from tree trunks at the height of 6–12 m (Diwakar & Balakrishnan, 2007a; Jain & Balakrishnan, 2011). Both sexes of *Gryllacropsis* sp. communicate acoustically using femoro‐abdominal stridulation (Diwakar & Balakrishnan, 2006). Males produce four‐syllable chirping calls, whereas female calls produce two‐syllable chirps either singly or in groups. Further, it is very difficult to differentiate between male and female Indian wetas in the field as females lack an external ovipositor similar to *Hemiandrus* sp. as reported by Gwynne (2004). The images of external genitalia of female and male under stereo zoom microscope (magnification 18×; Olympus SZX7) are shown in Figure 2. Females can only be distinguished acoustically in the field as females have two‐syllable calls.

In New Zealand, wetas have been reported to occupy a wide range of habitats from tree canopies, to the ground and caves (Gibbs, 1998). Most notably, they are reported to utilize preformed tree cavities, referred to as a “gallery” (Field, 2001). These shelters serve as a mating site for the males, as well as a place for mating and escaping predators (Field & Sandlant, 1983; Kelly, 2004). Choosing a habitat of suitable quality would increase not only the overall survival probability, by providing better foraging opportunities and protection from predators but also the reproductive success (Switzer, 1997). Thus, the present study attempted to investigate plant associations in *Gryllacropsis* sp. as certain plant species may be more suited to serve as food or serve as refuges against predators.

A previous study by Sinclair et al. (2001) has shown that seasons were important in determining the presence of *Hemideina maori*. Adverse climatic conditions may cause a reduction in activity and thus reduce population size (Cárdenas et al., 2017). The influence of climatic variables or seasonal shifts in calling activity has also been studied in sister groups of katydids (Tettigoniidae) and field crickets (Gryllidae) (Franklin et al., 2009; Fulton, 1951), but no studies have been made on this group from tropical forests in India.

Studies investigating sexual dimorphism in New Zealand wetas have shown that females are larger than males (Chappell et al., 2014; Gibbs, 1998; Kelly et al., 2008). In these species, females differ from males morphologically due to the presence of a long ovipositor and prominent size (Chappell et al., 2014; Watts et al., 2013). Thus, in the case of Indian weta, we quantified morphometric differences in body characteristics between males and females to identify other external characteristics that could be used to identify sex.

The life histories and biologies of anostostomatid orthopteran insects are only known from New Zealand species, and hence, understanding host plant association, calling activity, and sexual dimorphism will be important in outlining the biology of an Indian species. The objectives of the study thus were to (a) assess the host plant association, (b) investigate calling activity across months, and (c) investigate sexual dimorphism between sexes based on morphometric characteristics in the Indian weta *Gryllacropsis* sp. We tested the hypothesis that calling activity of Indian weta is uniformly distributed throughout the year and tested sexual dimorphism between males and females using multivariate analysis.

## 2 METHODS

### 2.1 Study system

The taxon was first described by Walker (1870) as *Gryllacropsis magniceps*. Another description of the taxon was given by Brunner von Wattenwyl (1888) as *Gryllacropsis perturbans*, which was later revised as a synonym for *G. magniceps* by Kirby (1906).0020The holotype used for description of *G. magniceps/G. perturbans* is a nymph described as a “male.” Females of the Indian weta lack external ovipositor, and there could be a possibility that the type specimen is a female nymph. Due to inability of verification with the type specimen, we have refrained from using the specific name in the study. The authors believe that lack of taxonomic clarity on specific name should not be a hindrance for investigating behavior and ecology of the study system as there are no other reported species of *Gryllacropsis* from India. Also, species‐specific songs of *Gryllacropsis* species serve as reliable indicators for species identity and can be used to avoid confusion between different species (Diwakar & Balakrishnan, 2007b; Shaw, 1999).

### 2.2 Study site and period

The study was conducted in Bhagwan Mahavir Wildlife Sanctuary and Mollem National Park, Goa, India. The park occupies 0.05% of the total area of Western Ghats with vast diversity and is protected for conservation of flora (Datar & Lakshminarasimhan, 2013). The sanctuary is spread over 240 km^2^ and extends between 15°15″30′ to 15°29″30′N and 74°10″15′ to 74°20″15′E. Humidity ranges from 50% to 96%, and the area receives an average annual rainfall of 308 cm. The forest type includes montane rain forests, moist deciduous forests, and dry deciduous forests (Datar & Lakshminarasimhan, 2013; Jadhav & Pati, 2012).

The host plant association was studied from May 2015 to December 2017. Field sites were visited around new moon phase as wetas were reported to be influenced by the lunar cycle and were found to be more active during the new moon phase as compared to the full moon phase (Diwakar & Balakrishnan, 2006; McIntyre, 2001). The vegetation assessment was carried out during day time, and calling activity sampling was carried out during night. The calling activity was studied from May 2015 to December 2017. Field sites were always visited before sunset, however, and no calling was heard before 6:00 p.m. Weta individuals called when the habitat was sufficiently dark. Therefore, acoustic sampling was carried out between 6:30 and 10:00 p.m. in the evening when the calling activity was known to be at peak (Diwakar & Balakrishnan, 2006).

### 2.3 Host plant association

The tree trunks were visually scanned to detect the presence of any weta individuals. Trees were marked on which weta individuals were spotted by observers (first author and field assistants). Marking trees during night time was limited to 10 m on either side of the forest track as accessing forest beyond 10 meters was difficult due to uneven or mountainous terrain and high encounter rates with wild animals such as leopards, bisons, and sloth bears. Quadrats of size 20 m long × 10 m wide were laid using a measuring tape (length—30 m) around the marked trees on which wetas were observed. A rectangular quadrat was thus chosen in a way such that the quadrat was large enough to be representative of the vegetation being investigated as well as logistically feasible.

Vegetation data were noted from the quadrats by counting all trees and identifying species. Plant species richness and abundance were quantified across all the quadrats. The marked and unmarked trees were noted in a quadrat to calculate the proportional availability and use of resources/habitat by the wetas. A total of 159 independent quadrats were laid for the vegetation assessment. In order to quantify the host plant association of wetas, resource selection function (RSF) was calculated by following the protocol developed by Jain and Balakrishnan (2011) based on the proportional use and availability of resources. The proportional availability of tree species was first calculated as follows: Proportional availability of habitat = number of individuals of particular tree species (h)/total number of individuals of all tree species sampled in the study area.

For example: *Terminalia paniculata* = 641/1984 = 0.40.

Proportional availability was calculated for all tree species available in the study area.

The proportional use of habitat by wetas was calculated as follows: Proportional use of habitat by weta = no. of individuals of wetas sampled from a particular tree species (s)/total number of individuals of wetas sampled within the study site.

For example: *T. paniculata* = 195/491 = 0.32.

Proportional use was calculated for all tree species available in the study area.

Selection function (*w_h,s_*) for a tree species was calculated using the same equation used by Jain and Balakrishnan (2011).

*w_h,s_* = proportion used/proportion available

For example: *T. paniculata* = 0.40/0.32 = 1.23.

Selection function (*w_h,s_*) was calculated for all tree species available in the study area.

Final step of standardization gives a value on a scale of 0–1. Value closer to 0 represents no preference, while values closer to 1 represent preferred host plants. It was calculated as follows:

$$b_{h,s}=\frac{W_{h,s}}{\sum_{i=1}^{H}W_{h,s}}$$

For example: *T. paniculata* = 1.23/49.42 = 0.02.

The standardization was carried out for all tree species (i.e., H = 52) available in the study area.

### 2.4 Calling activity

Psychoacoustic sampling was carried out to estimate the number of calling wetas (Diwakar et al., 2007). In psychoacoustic sampling, adult wetas were tracked acoustically on either side of transects and noted by a trained listener (first author). Trees on which the calling adults were heard were marked by the field assistant. A transect of 500 m was walked per night. The National Park has long stretches of undivided forest, and 20 transects of 500 m length were chosen for the study. Psychoacoustic sampling was carried out for a total of 89 nights on these 20 transects across different months. Sampling frequency varied across months. Sampling was restricted to two to three nights in the months when the calls were not heard, or very few calls over the 500‐m transect were heard.

Environmental parameters including ambient temperature (accuracy ± 1°C) and relative humidity (accuracy ± 3%) were recorded with a pocket weather meter (Kestrel 4500). Moon phase, GPS points (Garmin eTrex 10, accuracy within 100 m), and time were also noted at the same time. Sampling was carried out throughout the year except in the month of April, June, and July. Sampling had to be skipped in the month of April due to high heat and humidity and in the month of June and July due to heavy rainfall during monsoon season. Although the sampling was limited to 75% of a year, we believe that calling activity in April and June–July would be similar to the observations in the other months (March and May of the summer season) and (August–September) of the monsoon season, respectively.

The temporal variation in the calling activity was analyzed through a circular statistical analysis in ORIANA 2.0 (Kovach Computing Services). Months were converted into 30° angles, and abundance of calling activity in each month was taken as frequencies of each month observed (Zar, 1999). We estimated a) the mean angle (*α*), which represents the mean time of the year; b) the circular standard deviation (*SD*) related to *α*; and c) the length of the mean vector (*r*), a measure of data concentration around the circle (year), ranging from 0 (scattered data) to 1 (concentrated data on the same direction). The Rayleigh's uniformity test (*p* < .05) was used to indicate whether the calling activity was uniformly distributed and whether there is a significant mean angle or mean direction in calling activity of the Indian weta (Zar, 1999).

### 2.5 Sexual dimorphism

**FIGURE 3:**
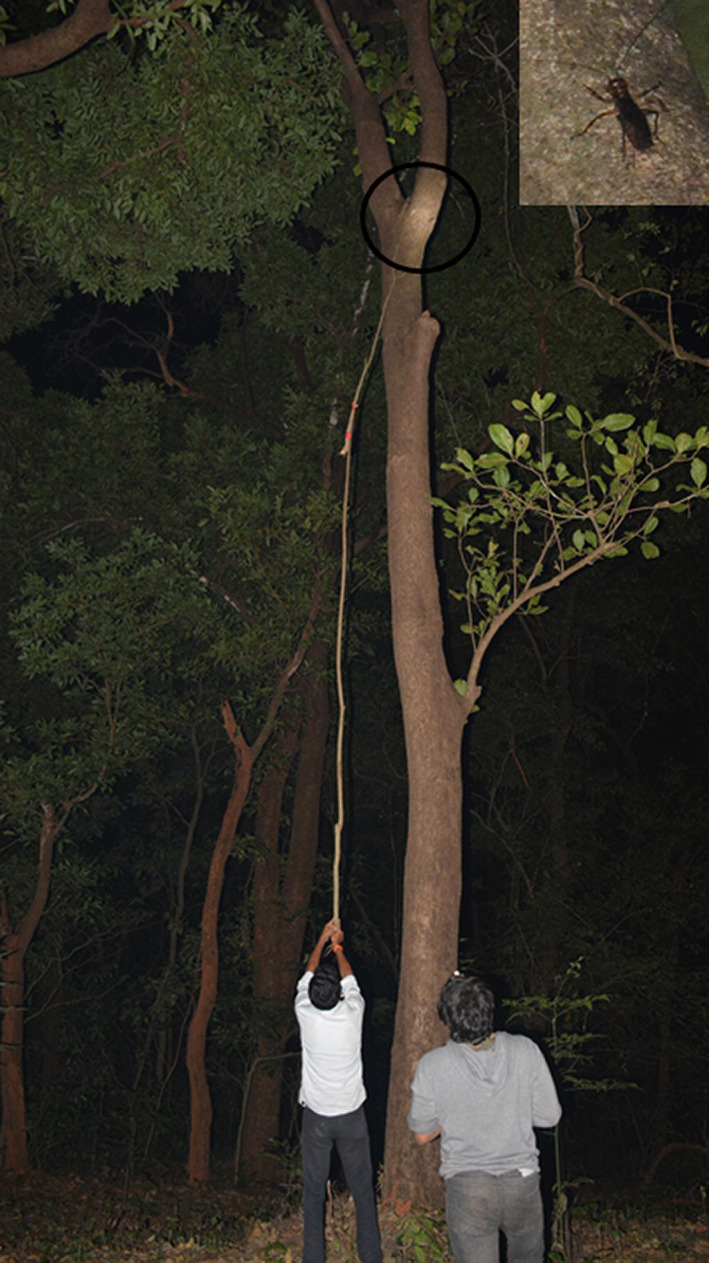
Image showing the *Gryllacropsis* sp. on a tree trunk at a height of 7 m. Black circle indicates the location of the weta on the tree trunk, and the inset shows the weta

Visual scanning was carried out to detect weta individuals from the base of the trunk to the canopy. Wetas were found roughly at the height of 7 m (Figure 3). Weta individuals were captured manually by (a) climbing trees or the neighboring tree in close vicinity, (b) using a ladder stacked against the trunk of the tree, and (c) using a stick to disturb the animal and catching the animal when they jumped down to escape. The captured individuals were placed in plastic boxes with perforated lids. Weta individuals were weighed inside the plastic box, and the weight of the box was subtracted to get the body weight of the individual.

**FIGURE 4:**
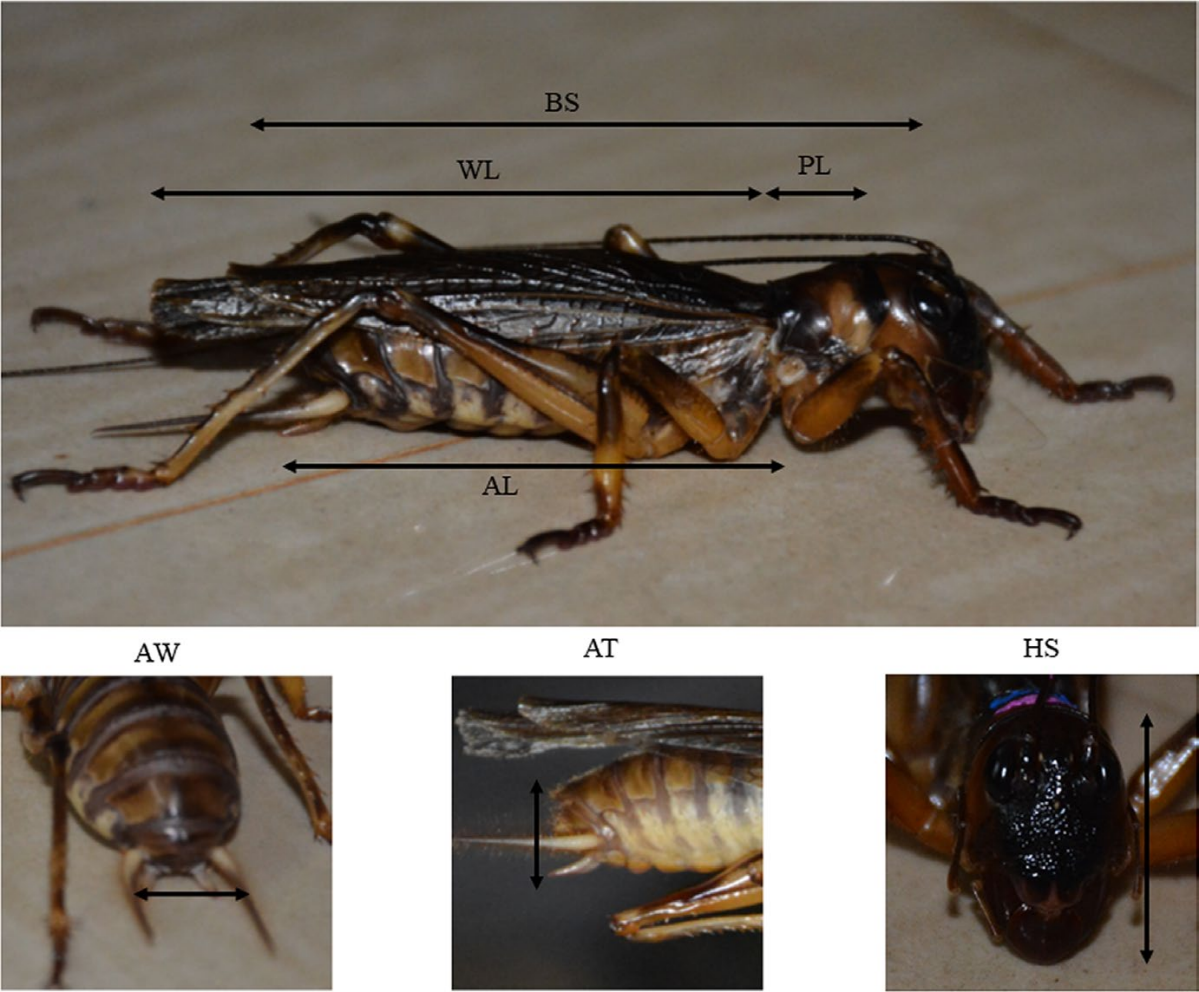
Morphometric parameters measured for the *Gryllacropsis* sp. AL, abdomen length; AT, abdomen thickness; AW, abdomen width; BS, body size; HS, head size; PL, pronotum length; WL, wing length

A total of 26 adults were captured for assessing the morphological measurements in males and females. The sex of each individual captured was determined by inspecting the subgenital plates. Each captured weta was weighed (BDLT Pocket Scale, 0.01–200 g) and measured using digital Vernier calipers (Aerospace Digimatic 0–150 mm). Nine morphometric characters were measured for each individual. These characters were body size (BS)—dorsal length from the head to the abdominal apex; wing length (WL)—length from the base of the wing to the apex; pronotum length (PL)—maximal distance from the anterior to posterior pronotal margins; head size (HS)—measured from anterior portion of the coronal suture at top of vertex till tip of mandible; abdomen length (AL)—length of the abdomen from 1st abdominal tergite to the last; abdomen width (AW)—measured the abdomen from side to side; abdomen thickness (AT)—the distance from base to the top of abdomen; abdomen volume—calculated by taking the product of AW, AT, and AL; and body weight—the total weight (Figure 4) (Chappell et al., 2014).

A discriminant function analysis (DFA) was performed using XLSTAT (Addinsoft, 2020) to differentiate between sexes using morphometric measurements. We used groups (males and females) as the dependent variable and nine morphological characters as independent variables. It was found that five of the nine variables, that is body size, wing length, pronotum length, abdomen width, and abdomen volume, had tolerance level of < 0.1 and variance inflation factor (VIF) values > 10. Hence, to avoid the problem of multicollinearity, DFA was conducted on four variables, viz. head size, abdomen length, abdomen thickness, and body weight. The number of canonical variables was one as the number of groups minus one (2 − 1 = 1).

## 3 RESULTS

### 3.1 Host plant association

**FIGURE 5:**
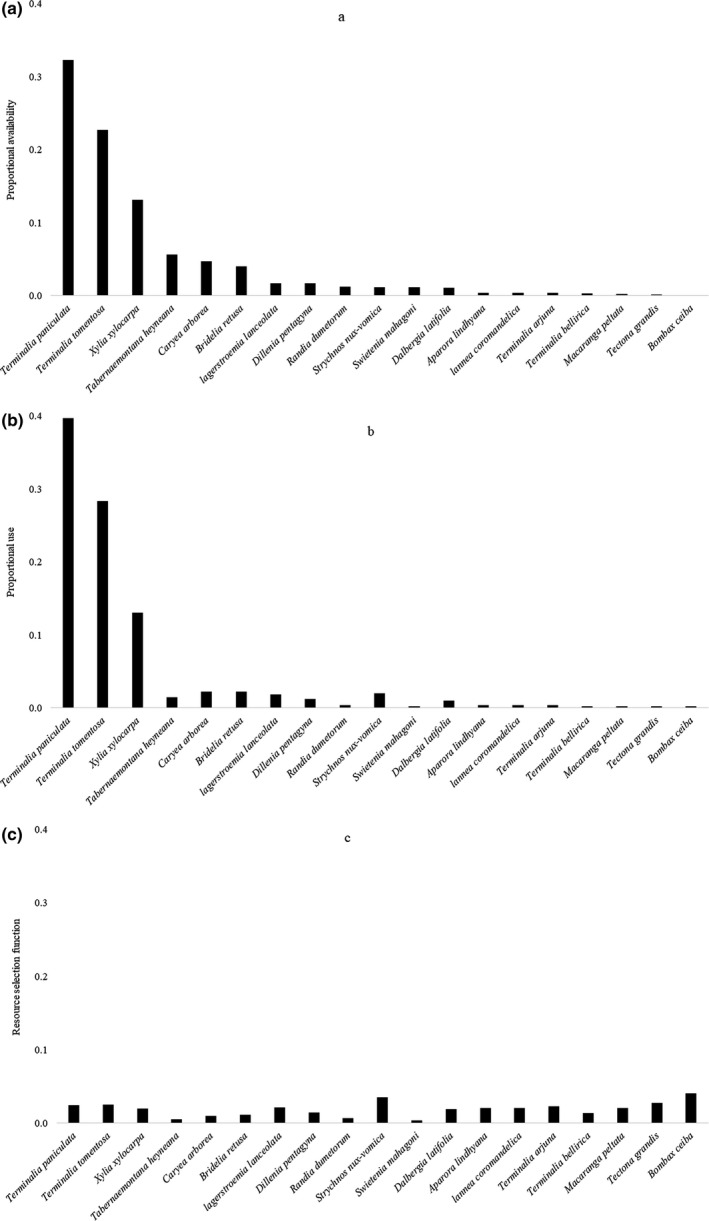
Bar graph showing (a) proportional availability of host plant, (b) proportional use of host plant, and (c) association of wetas to plant species using resource selection function

A total of 52 tree species were identified across all quadrats with a total of 1984 trees. Vegetation sampling showed that the most abundant tree species was *T. paniculata* with a total of 641 trees followed by *Terminalia tomentosa* (451 trees) and *Xylia xylocarpa* (261 trees) across all quadrats. The proportional availability of resources showed that the *T. paniculata* was most dominant (0.32) followed by *T. tomentosa* (0.23) in the study area. (Figure 5a). 334 out of 457 weta individuals were found to be calling from trunks of *Terminalia* sp. (Figure 5b). RSF values ranged from 0.02 to 0.08 for all species indicating no preference for any particular plant species by wetas (Figure 5c). We have only shown the truncated graphs as the data for the other species were too low.

### 3.2 Calling activity

**FIGURE 6:**
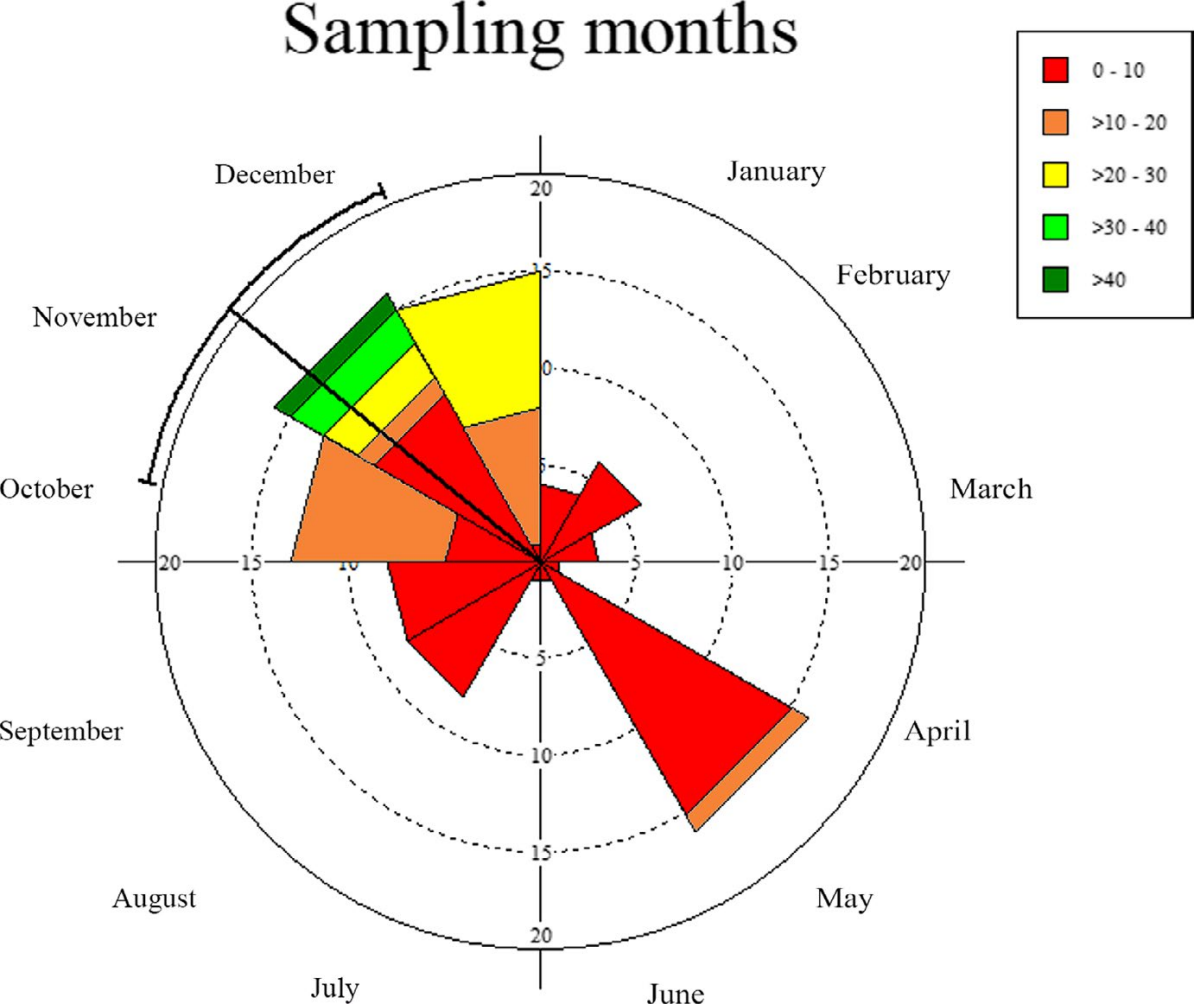
Rose diagram showing annual distribution of calling activity across different sampling months. The angles represent the months. The length of the mean vector (*r*) is a measure of concentration of data around the year. The values in the axes indicate the number of sampling nights, and the radius of wedges (shown in the legend) indicates the number of weta individuals heard in a month

A total of 728 calling individuals were counted across different sampling months. The circular statistical analysis indicated the concentration of calling activity between October and December (Figure 6). An arrow extending from circle's center toward the periphery indicated the mean angle and length of the mean vector (*r*) of 309.117° and 0.293, respectively, representing direction as well as measure of data concentration (Table 1). The mean angle of the calling activity was observed in the month of November (Rayleigh's test, *Z* = 7.90, *p* < .01). Lowest calling activity was recorded from early winter to summer (January to March). An isolated peak in the calling activity was recorded in the month of May, but the number of calling individuals heard was lower in May for the number of nights sampled.

**TABLE 1:** Results of circular statistics analysis for weta calling activity across different sampling months

Variables	Abundance
Observations (*n*)	92
Group width (number of groups)	30° (12)
Mean vector (*μ*)	309.117°
Mean group	November
Length of mean vector	0.293
Concentration	0.613
Circular variance	0.707
Circular standard deviation	89.766°
Standard error of mean	14.091°
Rayleigh's test (*Z*)	7.90
Rayleigh's test (*p*)	3.70E−04

### 3.3 Sexual dimorphism

**FIGURE 7:**
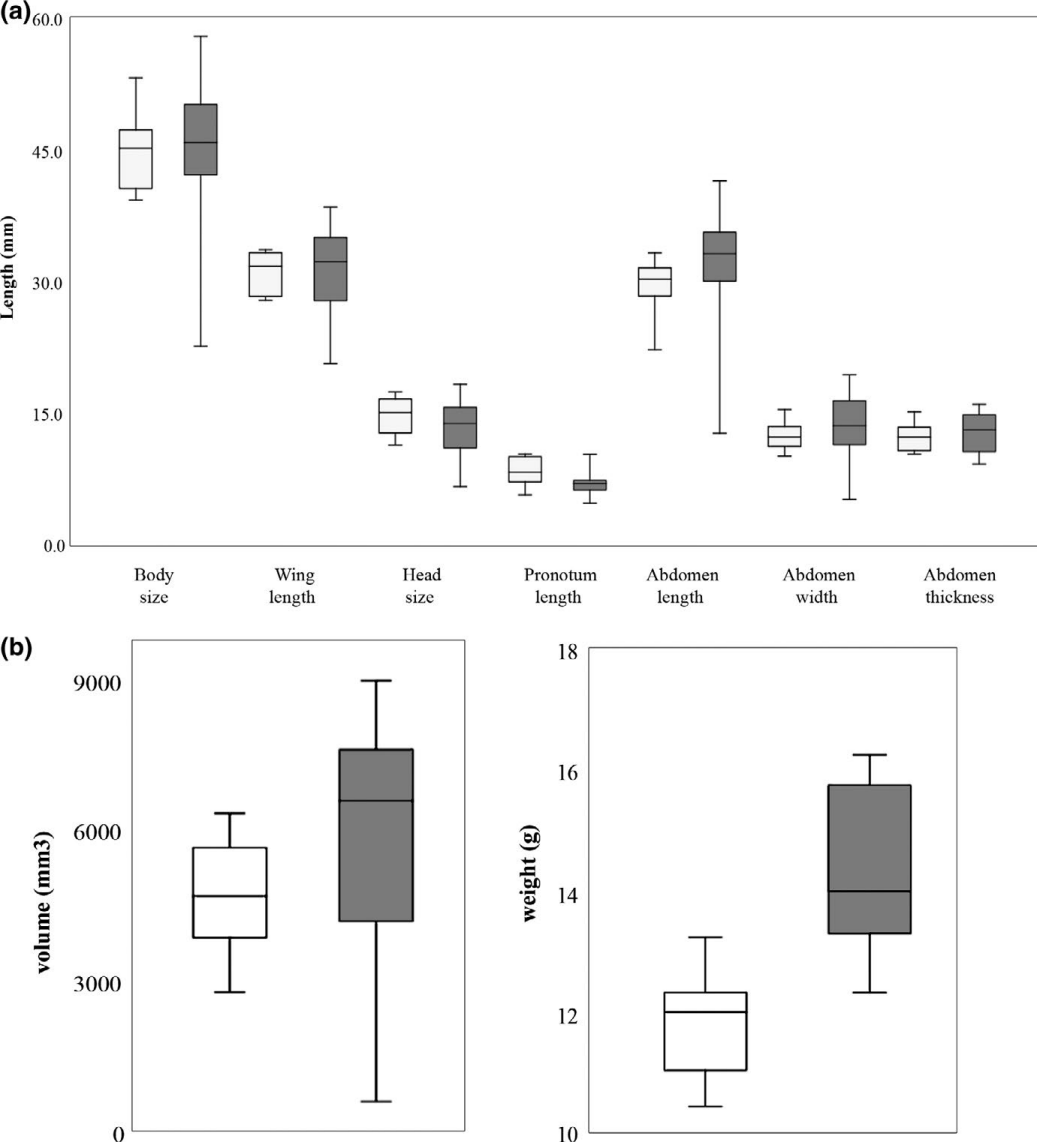
(a) Box plot showing comparisons of morphological measurements of adult females (*N* = 13) and males (*N* = 13) (b) Abdomen volume and body weight of both the sexes. Central line indicates median; box represents 1st and 3rd quartiles, and whiskers represent minimum and maximum values. Males are in white, and females are in gray color

**FIGURE 8:**
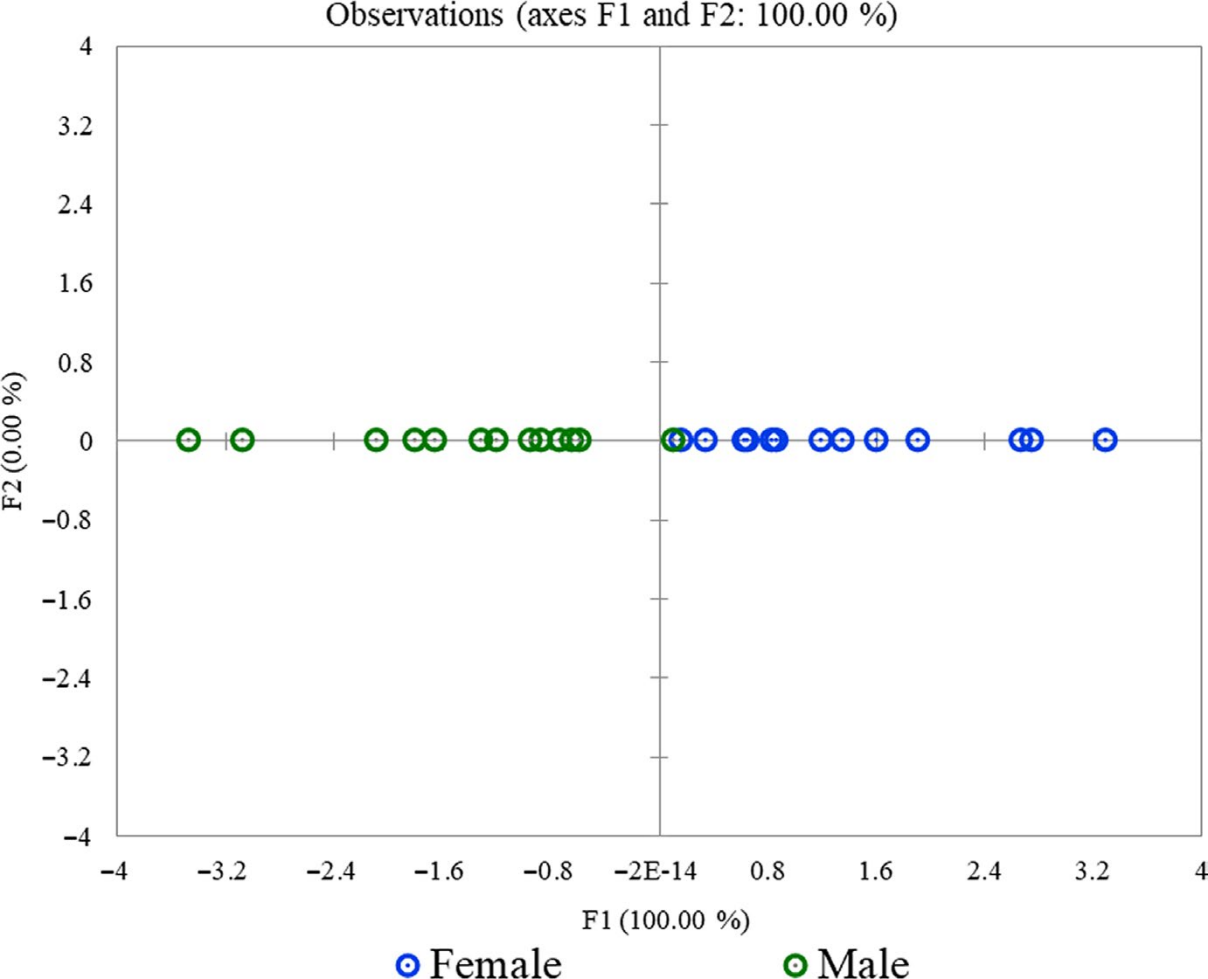
Linear discriminant analysis plot showing distribution of linear coefficients for females and males in the first canonical variate

Of the 26 adults captured, 13 were males and 13 were females. The median body weight of male was 12 g (range: 10.44–13.24 g) and female was 14 g (range: 12.32–16.26 g), respectively. The males had longer head size and pronotum length, whereas females were larger in wing length and abdomen parameters (Figure 7a). Females had longer abdomens and larger body weight as compared to the males (Figure 7b). Wilk's *λ* test (0.32 approx. *F* (4, 21) = 11.24 *p* < .0001) gave a highly significant result, giving a clear evidence that overall, these groups are meaningful. The contribution of body weight variable was significant (Wilk's *λ* = 0.4, *F* (1,24) = 36.57 *p* < .0001). The eigenvalue was 2.14 with 100% discrimination and classification accuracy of 96.15% (100% for females and 92.31% for males). Standardized canonical discriminant function coefficients for body weight were +1 (Table 2). A plot of the male and female individuals against their values for the canonical variables is shown in Figure 8. The plot shows a clear distinction between males on the left and the females on the right for the first canonical variate.

**TABLE 2:** Summary of the linear discriminant analysis for the four variables

Variables	Canonical discriminant function coefficient	Standardized canonical discriminant function coefficient	Classification function		Factor correlation	Unidimensional test of equality of the means			Multicollinearity statistics	
			Female	Male		*λ*	*F*	*p*‐value	Tolerance	VIF
Head size	−0.11	−0.30	1.18	1.5	−0.29	0.94	1.46	.24	0.56	1.78
Abdomen length	0.08	0.42	1.54	1.31	0.28	0.95	1.38	.25	0.65	1.53
Abdomen thickness	0.18	0.34	2.57	2.08	0.19	0.98	0.61	.44	0.47	2.14
Weight	0.89	0.99	15.63	13.13	0.94	0.4	36.57	<.0001	0.85	1.18
Intercept	−14.744		−162.52	−121.07						

Abbreviation: VIF, variance inflation factor.

## 4 DISCUSSION

There is limited information available for the Indian Weta *Gryllacropsis* sp. due to their camouflage with tree trunks, nocturnal activity, and arboreal and inaccessible dwelling habits. Indian wetas are endemic to Western Ghats, a biodiversity hotspot in India, and have peculiar characteristics such as both females and males of *Gryllacropsis* sp. communicate acoustically, individuals tend to prefer evergreen tree trunks as a microhabitat or calling sites, and female wetas lack an external ovipositor (Diwakar & Balakrishnan, 2006; Jain & Balakrishnan, 2011). Most ecological work on wetas has been mainly carried out on New Zealand wetas. The present study investigated host plant association, calling activity, and sexual dimorphism in the Indian Weta in its natural habitat to gain basic ecological information on the species. Our study indicated that wetas had no preference for any particular plant species. Peak calling activity was observed in the month of November. The body weight of male and female wetas proved important for distinguishing the sexes.

The present study investigated association of wetas to plant species using resource selection function. Most of the weta individuals were seen from *Terminalia* sp. followed by *Xylia* sp. as those were the dominant trees in the area. Wetas were also seen sucking sap from *Terminalia* sp. However, wetas did not show preference for any tree species. Previous study on microhabitat association of wetas reported that they call from the trunks (Diwakar & Balakrishnan, 2006). Also, *Gryllacropsis* sp. also showed 100% selection for tree trunk as preferred microhabitat or calling site (Jain & Balakrishnan, 2011). Studies have reported that *Deinacrida heteracantha* primarily inhabited *Cyathea dealbata* (43%) followed by *Rhopalostylis sapida* (16%) and least preferred *Geniostoma rupestre* (1%) (Gibbs & McIntyre, 1997; Watts & Thornburrow, 2011). *Hemideina* sp. was found to show no preference for the fruits of *Fuchsia* sp. over leaves of *Melicytus ramiflorus* (Wyman et al., 2011). Ecological divergence among populations that utilize different plant species can be analyzed by preference to feed, mate, or oviposit in association with the different hosts and performance in terms of growth, survivorship, and fecundity due to utilization of the different hosts (Funk et al., 2002). It will be interesting to examine the mechanisms that promote ecological diversification and reproductive isolation in wetas compared to other insects. Further, detailed studies of these wetas may shed light on nutrient balances and development rates by utilizing a wide range of food types (Bernays & Minkenberg, 1997; Raubenheimer & Simpson, 2003).

The present study observed calling activity of *Gryllacropsis* sp. in natural conditions. Wetas showed considerable variation in calling, with the highest activity during postmonsoon season. There was a low calling activity of adult wetas between January and March as compared to postmonsoon season. A relatively higher number of nymphs were observed during the dry months in February–March. A previous study has shown seasons were important in determining the presence of *H. maori* (Sinclair et al., 2001). Leisnham et al. (2003) reported the highest nymph activity of *H. maori* in New Zealand during summer. Watts et al. (2013) reported seasonal occurrence of *Deinacrida mahoenui* with two peaks of higher tracking rate in April and October. In the study of *Hemiandrus pallitarsis* by Gwynne (2004), mating period was reported during January. Calling response has been linked with weather conditions such as temperature, rainfall, and moon phase (Franklin et al., 2009; Moller, 1985; Thompson, 2013). Mating behavior and life cycle of the Indian weta remain to be investigated with reference to varying peaks of calling reported in the present study.

Our analysis on sexual dimorphism indicated that body weight was important for distinguishing between the sexes in the absence of an ovipositor. Also, few of the female body measurements had larger spread as compared to the males. This is in contrast to a study on Wellington Tree weta *Hemideina crassidens*, where males showed considerable variation in measured traits (Kelly, 2004). The larger body weight of females could be due to the presence of eggs as the females measured and dissected during the postmonsoon season (August–December) were found to be gravid. The mobility hypothesis suggests that female‐biased size dimorphism occurs because smaller males are favored as mates (Kelly et al., 2008). Previous studies have also reported the female‐biased sexual dimorphism in different species of New Zealand wetas such as *Deinacrida* sp. and *Hemiandrus* sp. (Chappell et al., 2014; Gibbs, 1998). It has also been reported that larger females are preferred by males as large size could be proportional to greater fecundity rate in insects in general (Honěk, 1993). Ovipositor length has been associated with maternal care in *H. pallitarsis* (Gwynne, 1995). It has been reported that species with long ovipositors do not show maternal care (Gwynne, 1995). It remains to be investigated if the pattern for maternal care holds true in the Indian *Gryllacropsis* sp. as well.

The absence of an ovipositor, larger body weight of females, polyphagy, and the calling behavior in *Gryllacropsis* sp. make it an important study system to address behavioral and ecological questions. The status of *Gryllacropsis* is unknown in the IUCN list. Further ecological studies can provide insights on abundance, distribution, and life history traits of these large invertebrates.

## CONFLICT OF INTEREST

The authors declare no conflict of interest.

## AUTHOR CONTRIBUTIONS

**Manisha Tomar:** Data curation (lead); formal analysis (equal); investigation (equal); methodology (equal); project administration (supporting); visualization (equal); writing – original draft (equal); writing – review and editing (equal). **Swati Diwakar:** Conceptualization (lead); data curation (supporting); formal analysis (equal); funding acquisition (lead); investigation (equal); methodology (equal); project administration (lead); supervision (lead); writing – original draft (equal); writing – review and editing (equal).

## Data Availability

The data is available at Dryad, Dataset, https://doi.org/10.5061/dryad.x69p8czgj Diwakar, Swati; Tomar, Manisha (2021), Data files for host plant association, calling activity and sexual dimorphism in an Indian weta, v4
